# Action of Dicumarol on Glucosamine-1-Phosphate Acetyltransferase of GlmU and *Mycobacterium tuberculosis*

**DOI:** 10.3389/fmicb.2019.01799

**Published:** 2019-08-20

**Authors:** Xiuyan Han, Changming Chen, Qiulong Yan, Liqiu Jia, Ayaz Taj, Yufang Ma

**Affiliations:** ^1^Department of Biochemistry and Molecular Biology, College of Basic Medical Sciences, Dalian Medical University, Dalian, China; ^2^Department of Microbiology, College of Basic Medical Sciences, Dalian Medical University, Dalian, China

**Keywords:** *Mycobacterium tuberculosis*, cell wall, GlmU, acetyltransferase, inhibitor, dicumarol

## Abstract

*Mycobacterium tuberculosis* is one of most pathogenic microorganisms in the world. Previously, the bifunctional enzyme GlmU with glucosamine-1-phosphate acetyltransferase activity and *N*-acetylglucosamine-1-phosphate uridyltransferase activity has been suggested as a potential drug target; therefore, discovering compounds targeting GlmU acetyltransferase is necessary. The natural products were tested for inhibition of GlmU acetyltransferase activity. We found that dicumarol exhibited inhibitory effects on GlmU acetyltransferase, with a concentration achieving a 50% inhibition (IC_50_) value of 4.608 μg/ml (13.7 μM). The inhibition kinetics indicated that dicumarol uncompetitively inhibited acetyl CoA and showed mixed-type inhibition for glucosamine-1-phosphate (GlcN-1-P). The activity of dicumarol against *M. tuberculosis* H37Ra was evaluated with a minimum inhibitory concentration (MIC) value of 6.25 μg/ml (18.55 μM) in the Alamar blue assay. Dicumarol also exhibited inhibitory effects on several clinically sensitive *M. tuberculosis* strains and drug-resistant strains, with a range of MIC value of 6.25 to >100 μg/ml. Dicumarol increased the sensitivity of anti-tuberculosis drugs (isoniazid and rifampicin) when dicumarol was present at a low concentration. The transcriptome and proteome data of *M. tuberculosis* H37Ra treated by dicumarol showed that the affected genes were associated with cell wall synthesis, DNA damage and repair, metabolic processes, and signal transduction. These results provided the mechanism of dicumarol inhibition against GlmU acetyltransferase and *M. tuberculosis* and also suggested that dicumarol is a potential candidate for TB treatment.

## Introduction

Natural products have a long history of use in the treatment of different diseases in China. Scientists working with herbs have isolated and identified many active components, which can explain many of their claimed therapeutic effects. Natural products, an important source of medicines, have been reported to possess anti-*Mycobacterium tuberculosis* activity (Cragg et al., 1997; Newton et al., 2000; Bunalema et al., 2017) due to their potential bioavailability and bio-permeability (Copp and Pearce, 2007; Dashti et al., 2014; Jyoti et al., 2015; Mehra et al., 2016). The extract of *Euphorbia fischeriana*, called “Jie-He-Ling” in China, has been employed clinically for the treatment of pulmonary tuberculosis. The isolated diterpenoids from *E. fischeriana* exhibited growth inhibition of *Mycobacterium smegmatis* (Wang et al., 2017). Therefore, we attempted to screen inhibitors of *M. tuberculosis* GlmU acetyltransferase from the compounds isolated from *E. fischeriana* and other herbs using a colorimetric assay for GlmU acetyltransferase, which was established in our laboratory (Zhou et al., 2011). We have not identified compounds from *E. fischeriana* targeting *M. tuberculosis* GlmU so far.

Tuberculosis (TB), caused by *M. tuberculosis*, is the ninth leading cause of death worldwide (Panicker et al., 2018). In 2017, 10 million people were infected with TB, including 5.8 million men, 3.2 million women, and 1 million children (under 15) (World Health Organization, 2018). It is still severe because of widespread multi-drug-resistant (MDR) strains and extensively drug-resistant (XDR) strains. The emergence of some drug-resistant strains is mainly due to mutations in the targets of antibiotics, the degradation or modification of antibiotics in the bacterial cell (Welch et al., 2005; Blondiaux et al., 2017), the overexpression of efflux pumps proteins and the change of porin in the cell wall to reduce the uptake of antibiotics (Hegde et al., 2001; Nikaido, 2003), some proteins involved in the antibiotic modulation/neutralization (Lata et al., 2015),the role of iron in imparting resistance to drugs (Kumar et al., 2013), and alteration of ribosomal RNA and small ribosomal subunit protein binding sites (Hegde et al., 2001). The bioinformatic analysis of some uncharacterized proteins might predict their interactive partners that were involved in various pathways of *M. tuberculosis.* These uncharacterized proteins were reported as potentially unknown mechanism of drug resistance (Sharma and Bisht, 2017). Therefore, there is an urgent medical need for new anti-*M. tuberculosis* agents or poly-therapeutic approaches to treat TB.

**FIGURE 1:**
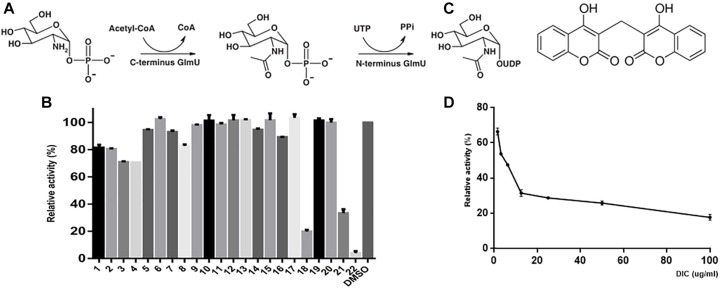
GlmU acetyltransferase assay and inhibitor screening. The first reaction is catalyzed by the acetyltransferase activity at the C-terminal domain, and the second reaction is catalyzed by uridylyltransferase at the N-terminal domain of GlmU **(A)**. The compounds were tested to screen the inhibitors of GlmU acetyltransferase activity **(B)**. The structure of dicumarol **(C)**. The best hit was compound 22 (dicumarol) with an inhibition >80%. The inhibitory effect of dicumarol on the growth of *M. tuberculosis* H37Ra **(D)**.

The cell wall of *M. tuberculosis* is incredibly complex, and the core structure consists of mycolic acids (MA), polysaccharide arabinogalactan (AG), and peptidoglycan (PG). AG attached to the PG through a disaccharide linker (D-*N*-GlcNAc-L-rhamnose) (Lee et al., 2006; Zhang et al., 2008). GlmU is a bifunctional enzyme with glucosamine-1-phosphate acetyltransferase activity at its C-terminus domain and *N*-acetylglucosamine-1-phosphate uridylyltransferase activity at its *N*-terminus domain. It catalyzes the two sequential steps to synthesize the glycosyl donor UDP-*N*-acetylglucosamine (UDP-GlcNAc) (Green et al., 2012) (Figure 1A), an intermediate in the synthesis of PG and its disaccharide linker during cell wall synthesis (Lee et al., 2006; Zhang et al., 2008). Previous reports show that GlmU is required for the integrity of the mycobacterial cell wall and essential to mycobacterial survival (Mcneil and Brennan, 1991; Sassetti et al., 2003; Rani et al., 2015). Therefore, GlmU is a novel and potential target for developing new anti-TB drugs. Therefore, we tried to screen and identify a compound with inhibitory effect on *M. tuberculosis* GlmU acetyltransferase activity. Dicumarol (3,3′-methylene-bis-4-hydroxycoumarin) was found to be an effective inhibitor for GlmU acetyltransferase. Dicumarol is a derivative of coumarin, and its structure is shown in Figure 1C. Then, the inhibitory manner of dicumarol was analyzed.

## Materials and Methods

### Bacterial Strains and Growth Conditions

*Mycobacterium tuberculosis* H37Ra (ATCC 25177) was growing at 37°C in Middlebrook 7H9 liquid broth supplemented with 10% ADC (albumin dextrose catalase complex), 0.4% glycerol, and 0.05% Tween 80 for drug susceptibility testing, infection assay, permeability assay, and transcriptome and proteome assays. Middlebrook 7H11 agar medium supplemented with 10% ADC and 0.4% glycerol was used for colony-forming unit (CFU) assay.

### Compounds and Drugs

The natural compounds were isolated from *E. fischeriana* and provided by the Pharmacy College of Dalian Medical University. The first-line anti-TB drugs [isoniazid (INH) and rifampicin (RFP)], cumarin, berberine, etc., were obtained from Shanghai Aladdin Bio-Chem Technology, and dicumarol was obtained from Sigma-Aldrich. The compounds and drugs were dissolved in DMSO at 10 mg/ml, while dicumarol was dissolved in DMSO at 4 mg/ml as stock solutions. Then, working solutions were prepared by diluting the stock solutions with 7H9 broth.

### Inhibitor Screening and IC_50_ Measurements

The *E. coli* BL21 (DE3)/pET16b-Mtb *glmU* strain stored in our laboratory was used to prepare the GlmU protein (Zhang et al., 2008). A colorimetric assay was performed to screen inhibitors for GlmU acetyltransferase. The product of GlmU acetyltransferase, CoA, reacted with DTNB [5,5′-dithiobis (2-nitrobenzoic acid)] and formed a yellow-colored molecular TNB^2–^, which could be detected at 405 nm (Zhou et al., 2011). Briefly, enzymatic reactions were performed in a 50-μl reaction solution containing 5 mM MgCl_2_, 50 mM Tris–HCl (pH 7.5), 0.4 mM acetyl CoA, 0.4 mM GlcN-1-P, and purified GlmU (0.04 μg) in 96-well plates at 37°C for 5 min. Reactions were terminated with 50 μl of 6 M guanidine hydrochloride and 50 μl of Ellman’s reagent [0.2 mM DTNB and 1 mM EDTA in 50 mM Tris–HCl (pH 7.5)] at room temperature for 5 min. The absorbance was measured at a wavelength of 405 nm. The “control reaction” containing acetyl CoA and GlcN-1-P without GlmU was used to correct the error due to the instability of substrates. The compounds at different concentrations were added to enzymatic reaction for testing their inhibition on GlmU acetyltransferase. Percent inhibition was calculated as% inhibition = 100 [1−(*A*_405_−*A*_Min_)/(*A*_Max_−*A*_Min_)], where *A*_Min_ is the absorbance of the control group, *A*_405_ is the absorbance of the test reaction that contained inhibitor, and *A*_Max_ is the absorbance of the uninhibited reaction. The concentration achieving 50% inhibition (IC_50_) was calculated using GraphPad.

### Mode of Inhibition

The dicumarol inhibition mode against GlmU acetyltransferase activity was determined by the DTNB colorimetric assay. The reactions were performed for one substrate (0.02, 0.04, 0.08, 0.12, and 0.16 mM) by keeping a constant concentration of the other substrate (0.4 mM). In this way, apparent inhibition constants were analyzed at three different concentrations (0 × IC_50_, 1 × IC_50_, 2 × IC_50_) of dicumarol. The kinetic parameters (*K*_m_, *V*_m_, *K*_m_^app^, and *V*_m_^app^) of dicumarol were calculated for both substrates GlcN-1-P and acetyl CoA.

### Susceptibility Testing

The minimal inhibitory concentration (MIC) of dicumarol was determined against *M. tuberculosis* H37Ra, GlmU-overexpressed *M. tuberculosis* H37Ra (H37Ra/pVV2-*glmU*), *M. tuberculosis* possessing the control vector pVV2 (H37Ra/pVV2), and clinically isolated strains of *M. tuberculosis* in microplates using a microplate-based Alamar blue assay (MABA) as described previously with some modifications (Collins and Franzblau, 1997; Palomino et al., 2002). Briefly, *M. tuberculosis* H37Ra, H37Ra/pVV2-*glmU*, and H37Ra/pVV2 were collected in the log phase and diluted to 1 × 10^5^ bacteria per well in a microtiter plate. Dicumarol was serially diluted in 48-well plates at a range of 0 to 50 μg/ml. The total volume in each well was 500 μl, including bacteria and drug, in 7H9 broth. The growth group (including bacteria), positive group (including bacteria and kanamycin), and blank group (only 7H9 medium) were also included. The plate was wrapped with parafilm and incubated at 37°C. On the sixth day, the 200-μl resazurin working solution (62.5 μg/ml) was added to the growth group, and the plate was incubated for an additional 24–48 h. If the growth group became pink, resazurin solution was added to all groups. Visual MIC was defined as the lowest concentration of drug that prevented the color change from blue to pink. The absorbance at a wavelength of 590 nm was measured using a Multiscan Fc spectrometer (Thermo Scientific).

### Combined Treatment of Dicoumarol With Anti-tuberculosis Drugs

The combination of dicumarol with INH and RFP against *M. tuberculosis* H37Ra was tested using Alamar blue assay. The assay was performed by keeping the concentration of dicumarol at half MIC (3 μg/ml). Briefly, the inhibitory effect of INH at 6.25–100 μg/ml was measured with or without dicumarol at 3 μg/ml, and the inhibitory effect of RFP at 0.75–12 ng/ml was measured with or without 3 μg/ml dicumarol. After treatment for 24 h, the 200-μl resazurin working solution was added, and the color change from blue to pink was observed in the plate. The absorbance at a wavelength of 590 nm was measured.

### Macrophage Infection Assay for *M. tuberculosis* H37Ra

The GFP (green fluorescence protein) gene was cloned in the pCG76 vector to generate a recombinant plasmid pCG-GFP that was stored in our laboratory. The pCG-GFP was electroporated into *M. tuberculosis* H37Ra, resulting in *M. tuberculosis* H37Ra/pCG-GFP. The *M. tuberculosis* H37Ra/pCG-GFP strain was constructed to investigate the anti-*M. tuberculosis* activity of dicumarol in *M. tuberculosis* H37Ra-infected macrophages. The mouse macrophage cell line RAW264.7 was utilized for infection by *M. tuberculosis* H37Ra/pCG-GFP (Skinner et al., 1995; Mangalindan et al., 2000). RAW264.7 cells were cultured in Dulbecco’s Modified Eagle Medium (DMEM) supplemented with 10% fetal bovine serum (FBS) at 37°C and 5% CO_2_ in a humidified incubator. Sterile glass slides were placed on the bottom of each well of a 24-well plate. The RAW264.7 cells were then diluted to 10^6^ CFU/ml and cultured in 24-well plates. The *M. tuberculosis* H37Ra/pCG-GFP strain was grown in Middlebrook 7H9 broth to mid-log phase for infecting RAW264.7 cells at a multiplicity of infection of 10 (MOI 1:10). After incubation for 4 h, the infected cells were washed four times with PBS to remove the extracellular bacteria and then incubated in dicumarol-containing medium for 24 h. Infected-RAW264.7 cells without dicumarol treatment were used as a control. After the cells were washed three times with PBS (pH 7.4), the glass slides were removed and surface cells were fixed in 4% paraformaldehyde for 20 min and then washed three times with PBS (pH7.4). The cell nucleus was stained using fluoroshield with DAPI (Abcam). Three independent experiments were performed.

### CFU Assay

The CFU assay was performed to measure the change of intracellular bacterial burden in macrophages after treatment with dicumarol. The RAW264.7 cells were infected with *M. tuberculosis* H37Ra for 4 h and then washed with PBS (pH7.4) to remove extracellular bacteria. The infected cells were incubated with dicumarol (MIC and 2×MIC) for 24 h. After being washed with PBS (pH 7.4), the cells were lysed by 0.01% SDS. The lysate was diluted and plated on a Middlebrook 7H10 agar plate, incubated at 37°C for 3–4 weeks to determine the number of colonies.

### Cytotoxicity

Cell viability was evaluated using 3-(4,5-dimethyl-2-thiazolyl)-2,5-diphenyl-2-H-tetrazolium bromide (MTT) assay (Mosmann, 1983; Twentyman and Luscombe, 1987). Briefly, RAW264.7 cells were seeded on a 96-well-plate (10^5^ CFU/ml). After incubation for 6 h in a 37°C CO_2_ incubator, the cells became adherent. Then, the medium was discarded, and fresh DMEM medium including different concentrations of dicumarol (4×MIC, 2×MIC, MIC, 0.5×MIC) was added and incubated for 24 h in a 37°C CO_2_ incubator. The medium in the wells was removed and 20 μl MTT (5 mg/ml) was added to each well of the plate. After incubation for 4 h, MTT in the wells was removed, and 150 μl of DMSO was added into each well followed by incubation in the dark for 10 min. The absorbance at 595 nm was measured. Three independent MTT assays were performed.

### PI Membrane Permeability Assay

Cell membrane integrity is associated with cell morphology and antibiotic susceptibility. Propidium iodide (PI) is considered to only stain cells with irreparably damaged membranes (Yang et al., 2015). Therefore, the cell wall and membrane integrity of *M. tuberculosis* H37Ra after dicumarol treatment were checked using a PI membrane permeability assay.

Briefly, the log phase of *M. tuberculosis* H37Ra was added with dicumarol (2×MIC) in a flask for 24 h, while untreated *M. tuberculosis* H37Ra was used as a control. The bacteria were harvested by centrifugation at 3,000 × *g* for 10 min and washed with 0.9% NaCl solution. Then, the bacteria were stained in 250 μl of a 0.9% NaCl solution that included 2 μl of the PI dye (Sigma) for 15 min in the dark. After staining, the bacteria were centrifuged and washed twice with 0.9% NaCl solution. The pellets were resuspended in 0.9% NaCl solution. The bacteria with damaged membranes were stained with PI and visualized as a red color under fluorescence microscopy, while the intact bacterial cells were not stained.

### The Effect of Dicumarol on the Transcriptome

*Mycobacterium tuberculosis* H37Ra was exposed to 12 μg/ml (2×MIC) of dicumarol for 24 h, while the DMSO group was used as the solvent control. The bacterial cells were collected by centrifugation at 5000 × *g* for 10 min and washed three times using 10 mM Tris buffered saline (TBS, pH 6.8). Briefly, total RNA was isolated from dicumarol-treated or untreated bacteria. First, bacteria were ground in liquid nitrogen to rapidly lyse the cells. After grinding, RNAiso Plus was added to cells, and the lysate was transferred into an RNase-free Eppendorf tube and kept at room temperature for 5 min. The lysate was centrifuged at 12,000 × *g* for 5 min at 4°C and the supernatant was transferred into a new Eppendorf tube. Chloroform of 0.2 times the sample volume was added to the supernatant and kept at room temperature for 5 min, followed by centrifugation at 12,000 × *g* for 5 min at 4°C. The aqueous phase was transferred into a new Eppendorf tube, and isopropyl alcohol of 0.8 times the sample volume was added. After incubation at room temperature for 10 min, the sample was centrifuged at 12,000 × *g* for 10 min at 4°C. The supernatant was discarded, and the pellet was washed twice with 75% thanol. Precipitated RNA was resuspended in RNase-free water. The extracted RNA was confirmed by 1.5% (w/v) agarose gel electrophoresis, and its quality was inspected using an Agilent 2200 TapeStation (Agilent Technologies). Libraries were prepared for sequencing period. RNA-seq was performed using an Illumina high-throughput sequencing platform by Hiseq3000/Hiseq 2500 (Illumina). After comparison and normalization, the fold changes between the treatment and control groups was calculated, and further bioinformatics analyses were carried out to better understand the functions of regulated genes, such as Gene Ontology (GO) enrichment and Kyoto Encyclopedia of Genes and Genomes (KEGG) pathway analysis.

### The Effect of Dicumarol on the Proteome

Log-phase *M. tuberculosis* H37Ra was treated with 12 μg/ml (2×MIC) of dicumarol for 24 h. DMSO was used as the solvent control. The cultures were harvested by centrifugation at 5,000 × *g* for 10 min and washed three times using 10 mM Tris–HCl (pH 6.8). The cell pellets were stored at −80°C for two-dimensional electrophoresis (2-DE) and RNA isolation experiments.

For 2-DE experiments, the cell pellets were resuspended in Tris–HCl (pH 6.8) containing 1 mM phenylmethanesulfonyl fluoride (PMSF) for sonication (work 30 s, stop 30 s, power 40%, 15 min, sonics vibra-cell^T*M*^). The sample was centrifuged at 12,000 × *g* for 30 min to pellet the cellular debris. The supernatant was treated with a 2-D clean-up kit (GE Healthcare) following the manufacturer’s instructions for 2-DE sample preparation. The total volume of 125 μl was incubated with an immobilized pH gradient (IPG) strip (pH 4–7, 7 cm) including a 50-μg protein sample in hydrated solution and 1 mM dithiothreitol (DTT) for 12 h. The first-dimension isoelectric focusing was performed at 250 V for 30 min, 500 V for 30 min, 4000 V for 30 min, and 4,000 to 20,000 V. Then, focused IPG strips were equilibrated for 30 min with the buffer containing 8 M urea, 1.5 M Tris–HCl (pH 8.8), 30% glycerol, 0.2% BPB, and 2% SDS. For the second dimensional electrophoresis, the IPG strips were run on homogeneous 12% SDS-PAGE at 5 mA for 15 min, 50 V for 30 min, 70 V for 30 min, 90 V for 1.5 h, and 110 V until bromophenol blue reached the bottom of the gel. The gels were stained using a ProteoSilver Silver Stain Kit (Sigma). ImageMaster 2D Platinum 6.0 software was used for quantification of protein spots in the gels. The significantly different protein spots were manually excised from gels for further detection by MALDI-TOF/TOF MS.

For RNA isolation and q-PCR analysis, total RNA extraction was common to the transcriptome from dicumarol-treated or untreated bacteria. The RNA concentration was quantified using a Nanodrop spectrophotometer (Thermo Scientific). A total of 1 μg of RNA was reverse-transcribed to cDNA using a TransScript One-Step gDNA Removal and cDNA Synthesis SuperMix Kit. Reverse transcriptase quantitative PCR (RT-qPCR) was performed using a TransStart Top Green qPCR SuperMix kit according to the manufacturer’s instructions.

The *sigA* gene, a housekeeping gene, was used for normalization. The relative expression of mRNA was calculated by 2^–ΔΔ^^CT^.

## Results

### Dicumerol Was an Inhibitor of GlmU Acetyltransferase

The natural compounds were tested for inhibition of *M. tuberculosis* GlmU acetyltransferase activity using the 5,5′-dithiobis-(2-nitrobenzoic acid) (DTNB) colorimetric assay. Only dicumarol showed the best inhibitory effect on GlmU acetyltransferase (Figure 1B). Its inhibitory activity was evaluated with an IC_50_ value of 13.7 μM (Figure 1D). However, dicumarol was unable to inhibit GlmU uridyltransferase activity by the malachite green colorimetric assay (data not shown).

**FIGURE 2:**
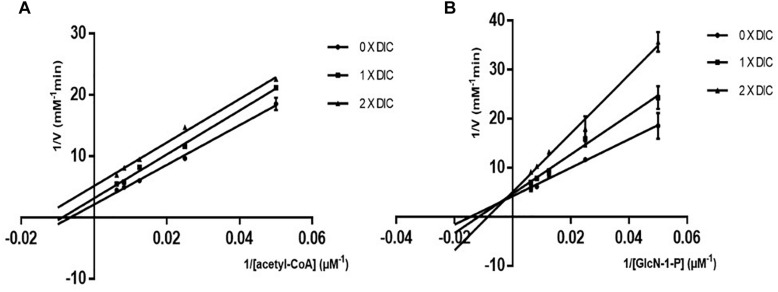
Mode of action of dicumarol inhibition. Parallel lines on a Lineweaver–Burke plot represented different dicumarol concentrations. The inhibition type was uncompetitive inhibition for acetyl CoA **(A)**. The lines of the different dicumarol concentrations merged at the second quadrant, suggesting a mixed type of inhibition for GlcN-1-P **(B)**.

To determine the inhibition mode of dicumarol on *M. tuberculosis* GlmU acetyltransferase activity, the apparent kinetic parameters were determined. The results showed that dicumarol was an uncompetitive inhibitor of acetyl CoA. In the Lineweaver–Burk plot (Figure 2A), the lines were displayed as parallel. The acetyl CoA *K*_m_^app^ and *V*_m_^app^ decreased with increasing dicumarol concentration (Table 1). Dicumarol exhibited mixed-type inhibition of GlcN-1-P, where the lines merged at the second quadrant in the Lineweaver–Burk plot (Figure 2B). The *K*_m_^app^ increased, and *V*_m_^app^ decreased with an increase in dicumarol concentration (Table 1). Molecular docking was used to predict the interaction of the compound and its target protein. It indicated that there was no direct interaction between dicumarol and the amino acid residues of the GlmU acetyltransferase domain without its substrates (data not shown). Since dicumarol was an uncompetitive inhibitor of acetyl CoA from kinetic analysis, the substrate acetyl CoA was required to bind with GlmU acetyltransferase first and dicumarol bound with the GlmU–acetyl CoA complex. Dicumarol showed a mixed inhibition for the second substrate glucosamine-1-phosphate (GlcN-1-P) of GlmU acetyltransferase. We proposed that binding of dicumarol to the GlmU–acetyl CoA complex affected binding of GlcN-1-P with the GlmU–acetyl CoA complex. This is why we did not get information about direct interaction of dicumarol with GlmU acetyltransferase without its substrates by docking analysis.

**TABLE 1:** *K*_m_^app^ and *V*_m_^app^ values of GlmU for acetyl CoA and GlcN-1-P.

**Concentration of dicumarol (μM)**	**Acetyl CoA *K*_m_^app^**	**Acetyl CoA *V*_m_^app^**	**GlcN-1-P *K*_m_^app^**	**GlcN-1-P *V*_m_^app^**
0	209.82	0.633 ± 0.1055	91.241	0.269 ± 0.019
13.7	94.967	0.286 ± 0.0478	106.972	0.199 ± 0.016
27.4	61.665	0.158 ± 0.0438	116.231	0.158 ± 0.013

### Dicumarol Effectively Inhibited the Growth of *M. tuberculosis*

**FIGURE 3:**
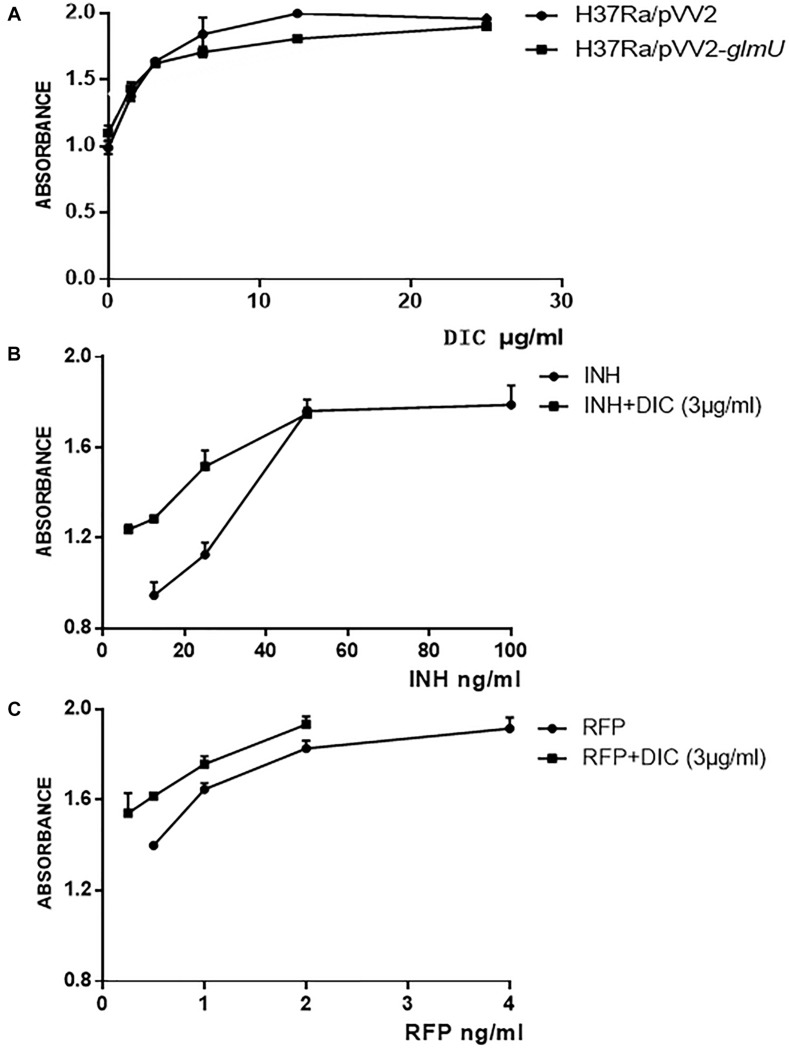
Susceptibility testing of dicumarol on bacterial growth. The inhibitory effect of dicumarol on the growth of *M. tuberculosis* H37Ra/pVV2 (∙) and *M. tuberculosis* H37Ra/pVV2-glmU (■) **(A)**. The absorbance was measured for the combination treatment of dicumarol and INH **(B)** or RFP **(C)** against *M. tuberculosis* H37Ra. INH/RFP treated alone (●), and INH/RFP combined with dicumarol (■).

The MIC was 12.5 μg/ml for *M. tuberculosis* H37Ra/pVV2-*glmU*, which was greater than the MIC of 6.25 μg/ml for *M. tuberculosis* H37Ra and *M. tuberculosis* H37Ra/pVV2 (Figure 3A and Table 2). This finding indicated that overexpressed GlmU in *M. tuberculosis* H37Ra/pVV2-*glmU* had a compensatory effect for dicumarol treatment. Thus, we thought that dicumarol acted on the GlmU target in *M. tuberculosis*. Moreover, the inhibitory capacity of dicumarol against four clinically sensitive and multi-drug-resistant strains of *M. tuberculosis* was also available with the range of MIC value of 6.25 to >100 μg/ml (Table 2). These results indicated that GlmU could be a target for *M. tuberculosis*, and dicumarol as an inhibitor of GlmU acetyltransferase has potential clinical application for TB treatment.

**TABLE 2:** MICs of dicumarol against different *M. tuberculosis* clinical strains.

**Strains**	**Number**	**MICs of dicumarol (μg/ml)**	**Strains**	**Number**	**MICs of dicumarol (μg/ml)**
H37Ra	ATCC 25177	6.25	MDR	15353	25
H37Ra/pVV2	–	6.25	MDR	15447	25
H37Ra/pVV2-*glmU*	–	12.5	MDR	18128	100
Sensitive	3326	12.5	MDR	16424	25
Sensitive	5103	25	MDR	18442	25
Sensitive	5148	50	MDR	18442	50
Sensitive	8246	25	MDR	22230	50
Sensitive	10307	12.5	MDR	30124	12.5
Sensitive	10308	>100	MDR	17174	100
Sensitive	11204	>100	MDR	17146	100
Sensitive	14301	>100	MDR	14165	6.25
Sensitive	13231	50	MDR	14220	50
Sensitive	15201	100	MDR	14306	50
MDR	16360	25	MDR	14315	50
MDR	15334	>100	MDR	13512	>100
MDR	15353	25			

*Dr. Hairong Huang at the National Clinical Laboratory on Tuberculosis, Beijing Key Laboratory on Drug-Resistant Tuberculosis Research, Beijing Chest Hospital, Capital Medical University, Beijing, China, tested dicumarol inhibition of *M. tuberculosis* clinical isolates.*

### Combined Treatment of Dicoumarol and INH/RFP Had a Cooperative Interaction

The combined treatment of dicumarol with anti-tuberculosis drugs against *M. tuberculosis* H37Ra was determined by the Alamar blue method. When dicumarol at a half MIC level (3 μg/ml) was combined with INH or RFP, the inhibitory activity of INH or RFP against an *M. tuberculosis* H37Ra strain was increased. From the absorbance curve (Figures 3B,C), INH at the concentration of 6.25–50 μg/ml or RFP at 0.25–2 μg/ml combined with dicumarol exhibited more obvious inhibitory effects on the growth of *M. tuberculosis* H37Ra compared with INH or RFP without dicumarol presence. When the concentration of INH or RFP was suitably increased, the anti-*M. tuberculosis* effects reached a maximum and were maintained at a constant level. These results indicated the cooperative interaction of dicumarol with INH or RFP. Therefore, a potential strategy for TB treatment is the administration of INH or RFP with dicoumarol as a combination therapy.

### Dicumarol Had Anti-*M. tuberculosis* Activity in Infected Macrophages

**FIGURE 4:**
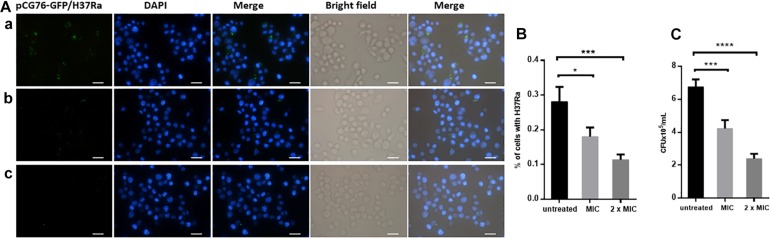
Anti-*M. tuberculosis* activity of dicumarol in *M. tuberculosis*-infected macrophages. Fluorescence microscope images of *M. tuberculosis* GFP-H37Ra-infected RAW264.7 cells (MOI = 10) following treatment with dicumarol (a = 0 μg/ml, b = 6.25 μg/ml, c = 12.5 μg/ml) for 24 h **(A)**. Blue: nuclei, Green: green fluorescent protein overexpressed in *M. tuberculosis* H37Ra/pCG-GFP. The percentage of bacteria in living cells was calculated by fluorescence microscope images **(B)**. Following dicumarol treatment to the infected-RAW264.7 cell, the RAW264.7 cells were lysed, and intracellular *M. tuberculosis* burden was determined by a CFU assay **(C)**. Data represent the mean ± SEM of triplicate samples (*n* = 3). ^*^p < 0.05, ^∗∗^*p* < 0.01, ^∗∗∗^*p* < 0.001, ^****^p < 0.0001 compared to that of untreated cell.

*Mycobacterium tuberculosis* is an intracellular bacterium that is cleared by macrophages. The green fluorescence from *M. tuberculosis* H37Ra/pCG-GFP within RAW264.7 cells was observed by fluorescence microscopy. After dicumarol treatment, the decreased number of intracellular bacteria in *M. tuberculosis* H37Ra/pCG-GFP-infected RAW264.7 cells were observed compared with that in untreated cells. The anti-*M. tuberculosis* activity of dicumarol increased in a concentration-dependent manner (Figures 4A,B). CFUs were determined at the beginning and end of the dicumarol treatment by lysing the macrophages and culturing the lysate on solid media. The CFU results confirmed the ability of dicumarol to inhibit intracellular *M. tuberculosis* (Figure 4C).

### Dicumarol Destroyed the Integrity of the Cell Wall and Membrane

**FIGURE 5:**
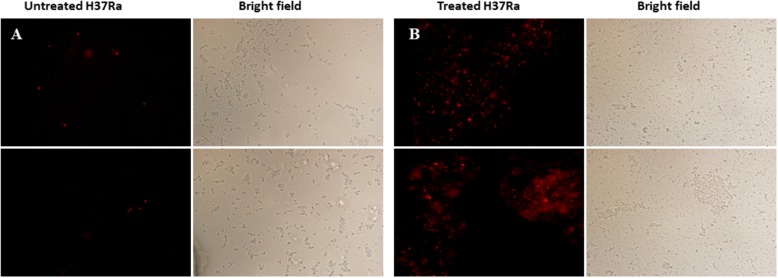
Fluorescence microscope images of membrane permeabilization. *M. tuberculosis* H37Ra was observed with red fluorescence of permeable PI when the integrity of the cell walls was destroyed. Untreated *M. tuberculosis* H37Ra **(A)** showed obvious red fluorescence than treated with dicumarol (12.5 μg/ml) **(B)**.

The cell wall and membrane are essential structures for the survival of all bacteria. PI, a cell-impermeable reagent, was used as an indicator to monitor the integrity of the *M. tuberculosis* cell wall after treatment with dicumarol. PI cannot enter cells with intact membranes, however, PI is permeable to damaged cell membranes. After PI enters a cell, it embeds within double-stranded DNA, and its red fluorescence can be observed by fluorescence microscopy. The red counts represent the PI-permeabilized live cells affected by dicumarol. Our results showed that the bacteria treated by dicumarol exhibited significantly higher PI permeability (red light) compared with that of untreated bacteria (Figures 5A,B), indicating that dicumarol led to a substantial cell wall and membrane destruction.

### Dicumarol Influenced mRNA Expression in *M. tuberculosis* H37Ra

**FIGURE 6:**
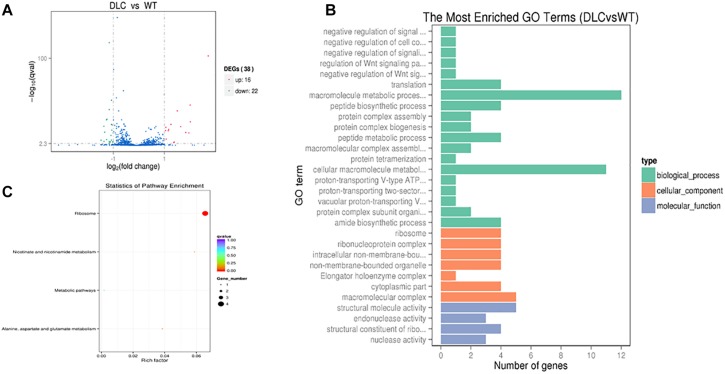
Transcriptional changes in *M. tuberculosis* H37Ra in response to dicumarol treatment. Differentially expressed genes (DEGs) were in *M. tuberculosis* H37Ra in the absence and presence of dicumarol (12 μg/ml) **(A)**. Gene Ontology (GO) analyzed the dicumarol-regulated genes **(B)**. The histogram sets for each functional category generated from DEGs according to the enriched gene counts. Kyoto Encyclopedia of Genes and Genomes (KEGG) pathway analyzed the dicumarol-regulated genes **(C)**. The list of DEGs is shown in Supplementary Tables S1, S2. WT, non-treatment; DLC, dicumarol treatment.

The influence of dicumarol on the transcriptome was analyzed using an RNA-seq method. A total of 38 significant differentially expressed genes (DEGs) were found with a| log2 (fold change)| > 1 (the log2 value of fold change (readcount of the dicumarol-treated group/readcount of the wild-type group) and *q* value <0.005 (*p* value is the hypothesis test probability and calculated by Poisson distribution mode; *q* value is corrected *p* value) (Supplementary Tables S1, S2). Volcano plots for the DEGs are shown in Figure 6A. However, the *glmU* gene was not found in the DEGs of the transcription level. These results indicated that dicumarol did not affect transcription of the *glmU* gene. GO and KEGG were used to analyze transcriptomic data to better understand the transcriptomic changes by dicumarol.

The GO functional classification of all the changed genes based on their biological processes, cellular components, and molecular functions is shown in Figure 6B. The classification results of GO terms showed that the largest protein group of DEGs was related to material metabolism and regulatory function, including macromolecule metabolic processes, peptide biosynthesis and metabolic processes, regulation of signaling, and regulation of cellular processes. For cellular components, DEGs are mainly involved in ribosomes, ribonucleoprotein complexes, non-membrane-bound organelles, and macromolecular complexes.

The results from KEGG analysis were involved in four pathways: (1) ribosomes (ID: mra03010); (2) nicotinate and nicotinamide metabolism (ID: mra00760); (3) alanine, aspartate, and glutamate metabolism (ID: mra00250); and (4) metabolic pathways (ID: mra01100) (Figure 6C and Supplementary Figure S2). According to the alanine, aspartic acid, and glutamate metabolism in bacteria, Asp and Ala generate the oxaloacetic acid and pyruvic acid by transferring amino group to Glu. Pyruvic acid enters the oxidation decomposition and oxaloacetic acid directly enters the tricarboxylic acid cycle (TAC) involved in energy metabolism.

### Dicumarol Influenced Protein Expression in *M. tuberculosis* H37Ra

To investigate the correlation of DEGs in the transcriptome and proteins in the proteome, the proteome of *M. tuberculosis* H37Ra treated by dicumarol was analyzed using two-dimensional electrophoresis in combination with MALDI-TOF/TOF MS. Seven clearly defined spots were selected from the 2-DE gels (Supplementary Figure S1A), and four proteins from these were identified with important function by using MALDI-TOF/TOF MS (Table 3). The four proteins were putative benzoquinone methyltransferase with *S*-adenosylmethionine-dependent methyltransferase activity; MoxR, a member of the ATPase family, with regulatory function; thiol peroxidase (Tpx), an important *M. tuberculosis* redox protein belonging to the peroxiredoxin family; and a conserved hypothetical protein, putative glyoxalase MRA_0584.

**TABLE 3:** Proteins identified (2-DE gel) using MALDI-TOF/TOF MS.

**Gene name (*M. tuberculosis* H37Ra)**	**Gene name (*M. tuberculosis* H37Rv)**	**Protein name**	**Function**	**Accession No.**	**Protein MW (Da)**	**Protein score (C.I.%)**	**Densitometric ratio**
MRA_0567	Rv0560c	Putative benzoquinone methyltransferase	*S*-adenosylmethionine-dependent methyl transferase activity	ABQ72290.1	25,929.1	100	2.49
MRA_1489	Rv1479	Putative transcriptional regulatory protein MoxR1	ATPase activity ATP binding	ABQ73233.1	40,737.5	99.921	1.61
MRA_0584	Rv0577	Conserved hypothetical protein (putative glyoxylase CFP32)	Glyoxylase	ABQ72307.1	27,325.4	100	4.87
MRA_1942	Rv1932	Thiol peroxidase	Thioredoxin peroxidase activity	ABQ73701.1	16,885.5	95.673	3.36

The mRNA expression of the four proteins detected with 2-DE-MALDI-TOF-MS and GlmU was analyzed using RT-qPCR. The primers used in this experiment are presented in Table 4, and the specificity of all primers was examined by NCBI primer-BLAST. The mRNA expression of the four identified proteins after normalization by *sigA* (a housekeeping gene in *M. tuberculosis* that was commonly used in internal reference gene) was similar to the changes at the protein level (Supplementary Figure S1B). Putative benzoquinone methyltransferase (MRA_0567) had a 5.838-fold change, and the other three genes had a small change. The data showed that GlmU (MRA_1026) did not change at the mRNA level, which confirmed the transcriptomic result that dicumarol did not affect the transcription of GlmU.

**TABLE 4:** Primers used for RT-qPCR.

**Genes**	**Forward primer (5′→3′)**	**Reverse primer (5′→ 3′)**
MRA_0567	TGTCGAACCTGCCGTCATAG	AGAACTGGCTCGGCATGAAG
MRA_1489	CCGAAGAAGAGCGCGAAATC	TTGTTGGCCGCTATCTCCTG
MRA_0584	CACGCTCATCTGGAACGAAC	CCGCAGCTATCTCCATGCTC
MRA_1942	CAATACCGTCGGTGAGCTAC	GTTCAGCAACACGGACTTAC
MRA_1026	GCGTTGACTTCGCGGATTTC	GATGATCCCTTCGGCTACGG
MRA_2731	CGGTGATTTCGTCTGGGATG	CCTTGCCGATCTGTTTGAGG

## Discussion

The cell wall of *M. tuberculosis* has been extensively studied due to the role in maintaining the structural integrity and impermeability to drugs or host immune system. Most studies on mycobacterial cell wall focused on the structures, biosynthesis, and functions of cell wall core containing mycolic acids, arabinogalactan, and peptidoglycan (Bhamidi et al., 2012). Therefore, more enzymes and proteins were identified as new targets to develop new anti-tuberculosis drugs (Grzegorzewicz et al., 2016; Abrahams and Besra, 2018). Bifunctional enzyme (GlmU) has glucosamine-1-phosphate acetyltransferase activity and *N*-acetylglucosamine-1-phosphate uridyltransferase (Rani and Khan, 2016). The glucosamine-1-phosphate acetyltransferase activity was not present in mammals and *N*-acetylglucosamine-1-phosphate uridyltransferase in bacteria was non-homologous with AGX1 and AGX2 in mammals (Mio et al., 1998; Peneff et al., 2001; Milewski et al., 2006). Therefore, GlmU has been utilized as a target to develop new antibiotics.

Different strategies have been utilized to find inhibitors on *M. tuberculosis* GlmU. Structure- and ligand-based computational models were developed to screen the drug-like compounds for the identification of probable inhibitors of *M. tuberculosis* GlmU (Mehra et al., 2016). An *in vitro* high-throughput bioassay was used to identify inhibitors of the acetyltransferase domain of *M. tuberculosis* GlmU (Rani et al., 2015). The quantitative structure–activity relationship (QSAR) and docking techniques were developed for predicting the inhibitory activity (IC_50_) of chemical compounds against *M. tuberculosis* GlmU (Singla et al., 2011). In our laboratory, we focus on inhibitors of GlmU acetyltransferase. The large number of compounds from different natural sources were screened for inhibitors of *M. tuberculosis* GlmU by using DTNB colorimetric assay and dicumarol exhibited effective inhibition on GlmU acetyltransferase. It was found that the diterpenoids from *E. fischeriana* were capable of inhibiting the growth of *M. smegmatis* (Wang et al., 2017) and *M. tuberculosis* H37Ra (unpublished data). However, the diterpenoids did not act on GlmU acetyltransferase. Here, we tested the effect of dicumarol on mycobacterial growth, the influenced mRNA and protein expression in *M. tuberculosis* H37Ra, the anti-*M. tuberculosis* activity in infected macrophages, as well as its cytotoxicity (Supplementary Figure S3).

Some research groups worked on different drugs or compounds against *M. tuberculosis*. Based on different study aims, different experiments using different concentrations of drugs or compounds were performed (Betts et al., 2003; Provvedi et al., 2009; Ballinger et al., 2019). Freiberg et al. mentioned that concentrations of antibiotics and treatment time should be chosen carefully for obtaining the expression profiles. The fractions to multiples of the MIC of an antibiotic for time frames covered the first minutes after compound exposure to up to more than one generation time (Freiberg et al., 2004). Dicumarol was firstly found with anti-*M. tuberculosis* activity in our laboratory. It is one of the derivatives of coumarin and is a naturally coumarin-based compound in spoiled sweet clover, which has been used as an oral anticoagulant drug owing to its ability to suppress prothrombin synthesis (Goth, 1945; Link, 1959). Many coumarin derivatives are biologically active and a number of them were studied for antimicrobial activity (Link, 1959). For drug susceptibility testing in this study, dicumarol was incubated with *M. tuberculosis* H37Ra at lag phase to determine the MIC. Since transcriptomic and proteomic analyses needed logarithmic phase bacteria, the more bacterial cells needed to be treated using a higher concentration of dicumarol than the MIC. The growth of *M. tuberculosis* is slow, and it takes 18–24 h for one generation. Therefore, we used 2×MIC to treat *M. tuberculosis* H37Ra for 24 h for the transcriptomic and proteomic analyses.

The transcriptome and proteome of bacteria were dynamic entities rapidly responding to the changes in environmental conditions. On one hand, the translational level was related to the target’s physiological function by the respective compounds. On the other hand, compounds also changed the expression of many genes not directly linked to the target’s function (Freiberg et al., 2004). UDP-GlcNAc is a donor for linker disaccharide and a precursor for peptidoglycan during biosynthesis of mycobacterial cell wall, which was catalyzed by three enzymes, glucosamine-6-phosphate synthase (GlmS), phosphoglucosamine mutase (GlmM), and bifunctional enzyme (GlmU). The first step reaction of the UDP-GlcNAc pathway is catalyzed by GlmS, which converts substrates fructose-6-phosphate and glutamine to products glucosamine-6-phosphate and glutamate. Fructose-6-phosphate is an important intermediate metabolite in glycolysis and regulates glycolysis by forming into fructose-1, 6-diphosphate and converting into other monosaccharides. Glutamine is a substrate for biosynthesis of protein and *de novo* biosynthesis of nucleotides. The bifunctional enzyme GlmU catalyzes the last two step reactions of the UDP-GlcNAc biosynthetic pathway. Once the GlmU activity is inhibited, the pathway of UDP-GlcNAc biosynthesis will be blocked and metabolism of carbohydrates and nucleotides might be influenced because of imbalance of intermediate metabolites. Therefore, the transcriptome and proteome of *M. tuberculosis* H37Ra treated with dicumarol were analyzed. A number of genes involved in DNA damage and repair at the transcriptomic level, including HNH endonuclease, DNA repair exonuclease, and DNA repair protein RadA, were found to be up-regulated. However, the down-regulated genes were involved in ribosomal proteins, including the 50S ribosome protein L4, the 50S ribosome protein L35, the 30S ribosome protein S11, and the 50S ribosome protein L36. Therefore, the genetic damage by dicumarol treatment may be due to an altered intracellular redox environment. From the results of the *M. tuberculosis* H37Ra proteome, thiol peroxidase (Tpx) was identified. Tpx is an important *M. tuberculosis* redox protein belonging to a peroxiredoxin family that plays a dominant role in anti-oxidant defense (Horst et al., 2010). It may have a role in the defense system against oxidative stress caused by toxic derivatives. This finding was consistent with the altered genes related to DNA damage and repair identified in the results of the transcriptome analysis.

In addition, other down-regulated genes were found in the transcriptome data as follows: KasA, a beta-ketoacyl-[acyl-carrier-protein] synthase II (MRA_RS11920) protein, which is involved in mycolic acid biosynthesis and survival of *M. tuberculosis* (Schiebel et al., 2013; Durairaj and Shanmughavel, 2019). Also identified was HspX, encoding a 16-kDa a-crystallin-like protein. The overexpression of a-crystallin in rBCG (recombinant attenuated live vaccine strain of *Mycobacterium bovis*) has previously been reported to protect aging bacteria against autolysis to some extent, due to its chaperone activity that stabilized the cell wall and intracellular structures (Hu et al., 2006). RadA, a DNA-dependent ATPase, was involved in the processing of recombination intermediates and played a role in repairing DNA breaks. In addition, it was reported that RadA inhibited c-di-AMP synthesis in *M. smegmatis*, and this inhibitory role is conserved in many bacteria. C-di-AMP is recognized as a ubiquitous second messenger involved in the regulation of bacterial signal transduction (Beam et al., 2002; Zhang and He, 2013). Benzoquinone methyltransferase was identified in both the transcriptome and proteome data. It is involved in many biological processes, such as cellular responses to iron ion starvation, methylation, hypoxia, and salicylic acid. Recently, it was reported that benzoquinone methyltransferase inactivated drugs by methylation, which resulted in bacterial resistance (Malone and Gordon, 2016; Warrier et al., 2016).

Dicumarol also had a high anti-*M. tuberculosis* activity against several *M. tuberculosis* clinical strains and inhibited the growth of *M. tuberculosis* H37Ra within RAW264.7 macrophages. In addition, combined treatment with dicoumarol and INH or RFP produced a cooperative effect against *M. tuberculosis* H37Ra, which reduces the MIC of each drug. Importantly, our study found that dicumarol had no obvious toxicity toward RAW264.7 macrophages. This finding suggests that dicumarol has a prospective use with clinical anti-TB drugs or use in the treatment of clinical multi-drug-resistant strains for a better therapeutic effect.

Overall, our study found that dicumarol inhibited GlmU acetyltransferase *in vitro* enzyme activity assay. The small-molecule dicumarol was capable of entering the bacterial cell and targeting GlmU, leading to inhibition of the UDP-GlcNAc biosynthetic pathway. The integrity of the cell wall and membrane was destroyed, and the intracellular homeostasis was disrupted. Therefore, dicumarol indirectly influenced the affected genes associated with DNA damage and repair, metabolic processes, and signal transduction. These data provide a more comprehensive understanding of the inhibitory mechanism of dicumarol against *M. tuberculosis*.

## Author Contributions

All authors have seen and approved the content and have contributed significantly to this work. YM, XH, and CC contributed to the conception and design. XH, CC, QY, LJ, and AT performed the experiments. XH, CC, and QY contributed to the data analysis and figure preparation. XH, YM, and CC were in charge of writing.

## Conflict of Interest Statement

The authors declare that the research was conducted in the absence of any commercial or financial relationships that could be construed as a potential conflict of interest.
