# Supplementary material for: Action of Dicumarol on Glucosamine-1-Phosphate Acetyltransferase of GlmU and Mycobacterium tuberculosis
Source: Front Microbiol. 2019 Aug 20;10:1799. doi: 10.3389/fmicb.2019.01799 (PMC6710349; doi:10.3389/fmicb.2019.01799)
Supplement: Supplementary file 1 [file Data_Sheet_1.pdf]

Supplementary Figures and Tables

A

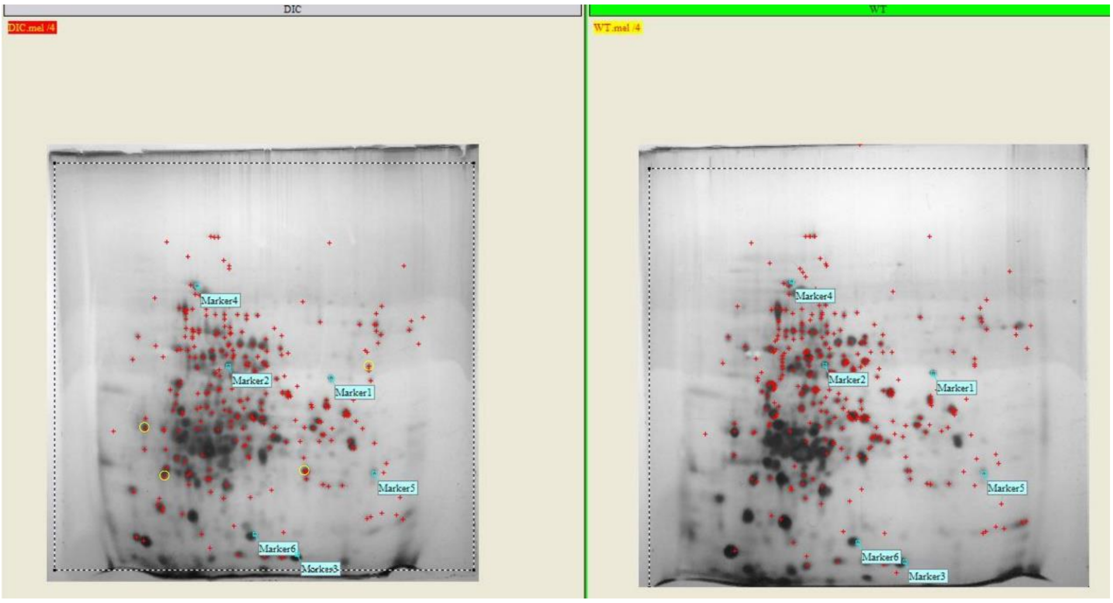

B

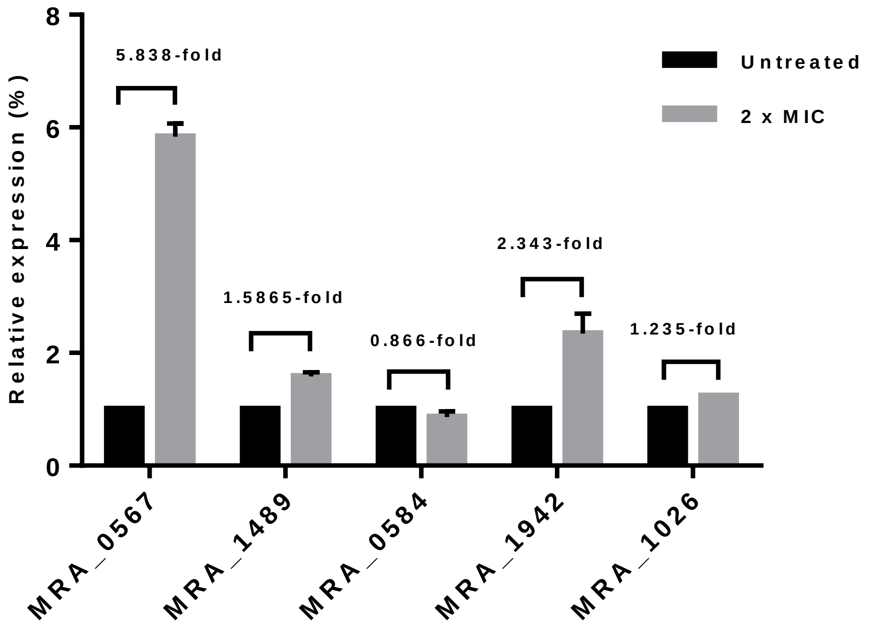

Figure S1. Comparison of the protein expression profiles. 2-DE images of *M.tb* H37Ra in the presence and absence of 12.5 μg/ml dicumarol. Circles indicated significantly altered spots that were identified by MALDI-TOF/TOF (A). Relative change of the proteins was confirmed at the

mRNA level by qRT-PCR (B). MRA_0567: MRA_0567: putative benzoquinone methyltransferase. MRA_1489: putative transcriptional regulatory protein MoxR1. MRA_0584: conserved hypothetical protein (putative glyoxylase CFP32). MRA_1942: thiol peroxidase. MRA_1026: *glmU*.

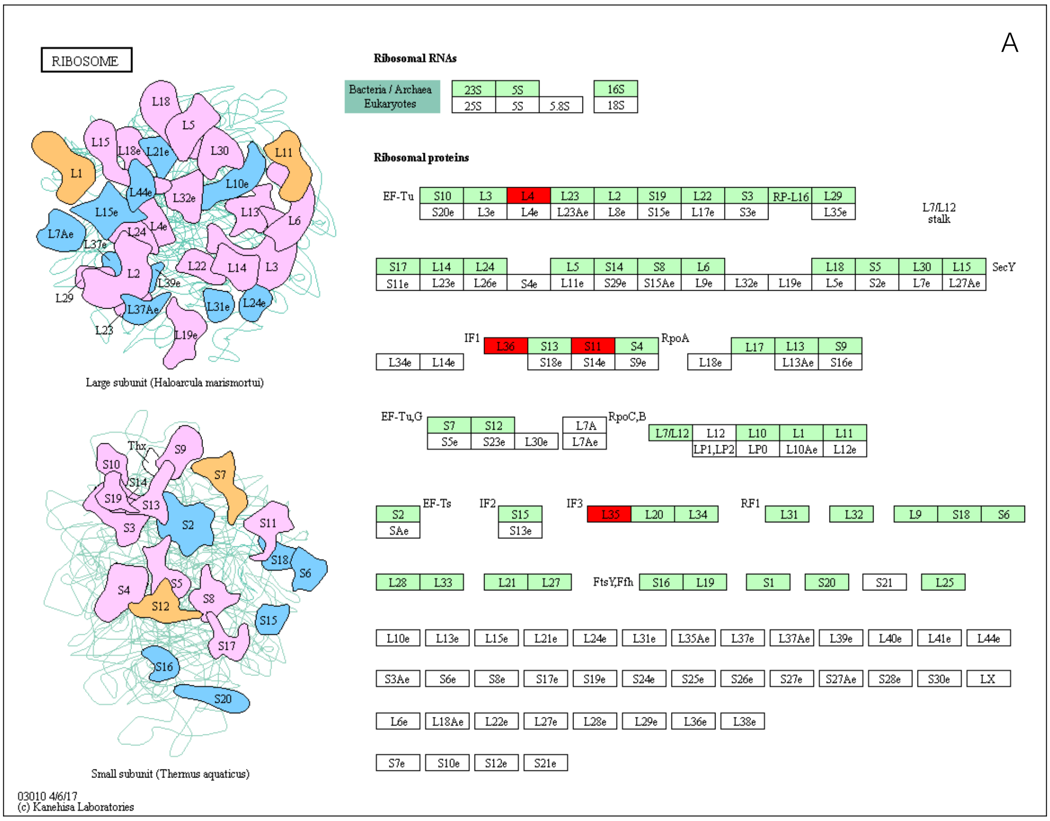

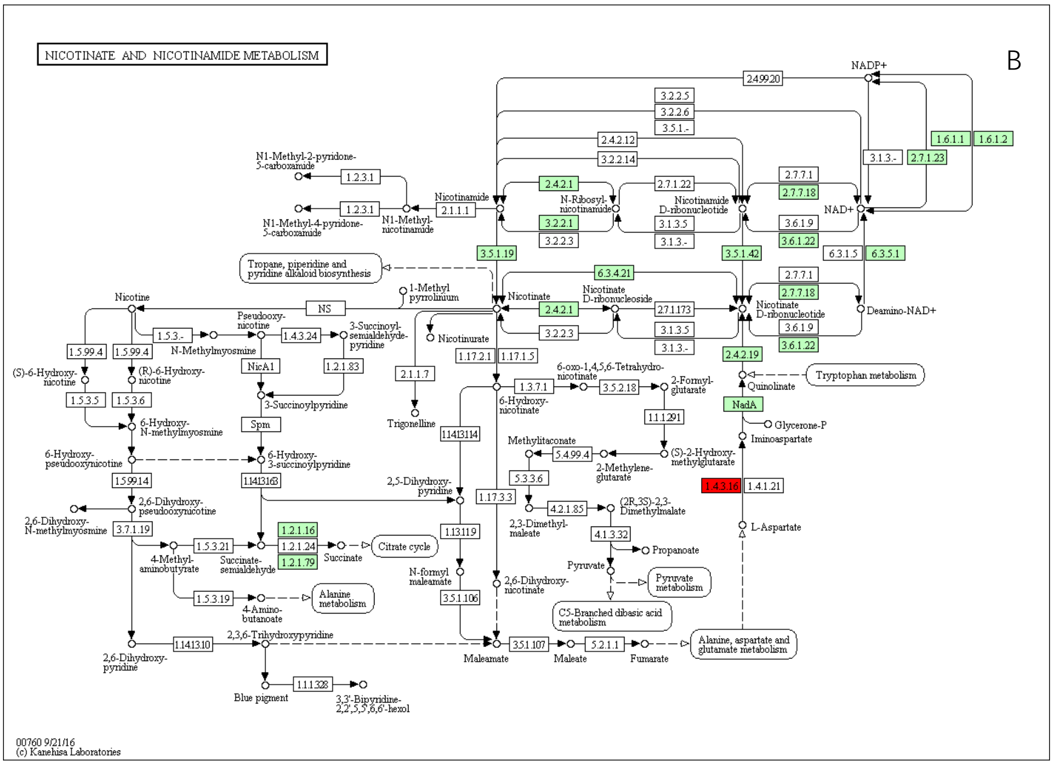

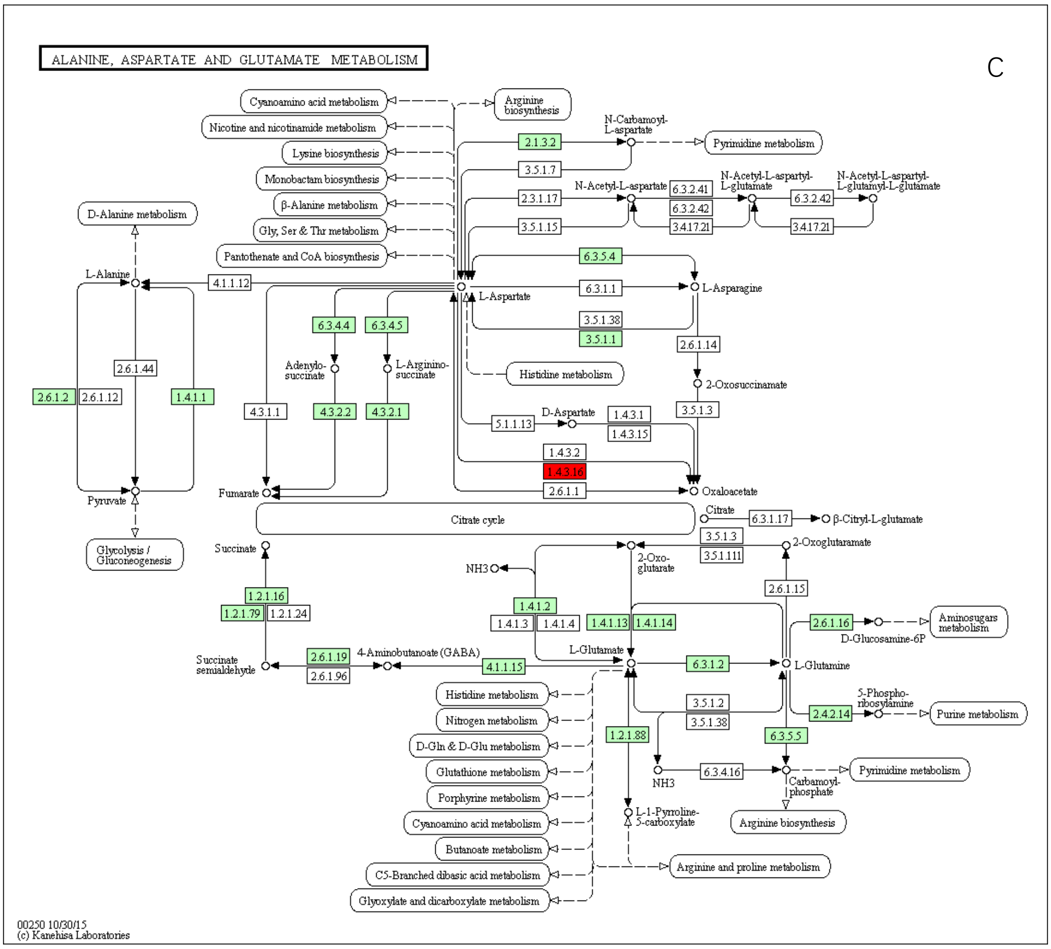

Figure S2. KEGG pathway analysis of DEGs. DEGs were highly enriched for ribosome (A),

nicotinate and nicotinamide metabolism (B), alanine, aspartate and glutamate metabolism pathway (C).

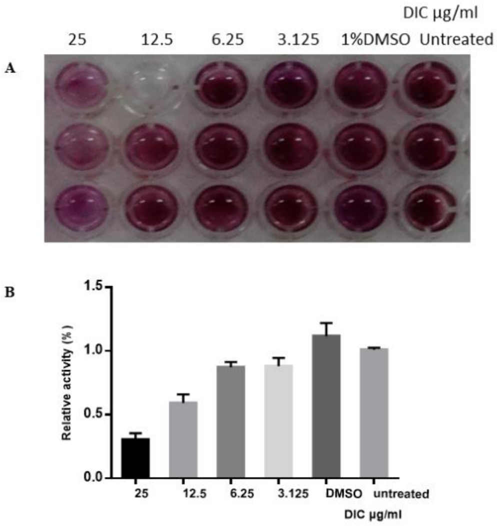

Figure S3. Cytotoxicity assay. The viability of RAW264.7 cells treated with different concentrations of dicumarol was compared to that of untreated cells by MTT method. There was not obvious cytotoxicity at the MIC (6.25 μg/ml) concentration of dicumarol.

Table S1. List of differentially up regulated genes

Gene_id	log2.Fold	pvalue	qvalue	Description
MRA_RS02940	2.7185	3.14E-106	1.99E-103	benzoquinone methyltransferase
MRA_RS03170	1.1707	5.52E-21	3.51E-19	IS607 family transposase IS1536
MRA_RS03175	2.0122	1.46E-49	3.71E-47	transposase
MRA_RS06115	1.3654	3.60E-05	0.00037679	hypothetical protein
MRA_RS06770	1.2596	4.61E-27	5.33E-25	DNA repair exonuclease
MRA_RS08270	1.5066	1.01E-42	1.97E-40	transporter MmpL6

MRA_RS10275	1.3874	8.58E-06	0.00010293	hypothetical protein
MRA_RS10340	1.0714	2.00E-09	4.57E-08	SecB-like chaperone
MRA_RS10670	1.838	2.49 E-17	1.13E-15	HNH endonuclease
MRA_RS11615	1.9606	7.68E-17	3.31E-15	hypothetical protein
MRA_RS15830	1.1555	2.01E-19	1.04E-17	IS607 family transposase
MRA_RS16945	1	4.80E-08	8.90E-07	hypothetical protein
MRA_RS16950	2.0164	4.81E-30	6.80E-28	hypothetical protein
MRA_RS19015	1.0585	1.94E-23	1.45E-21	DNA repair protein RadA
MRA_RS21805	1.0451	1.10E-19	5.84E-18	hypothetical protein
Novel00022	1.6408	2.80E-24	2.23E-22	

pvalue is the hypothesis test probability and calculated by poisson distribution mode.

qvalue is corrected pvalue and qvalue < 0.05 this function is an enrichment item.

Table S2. List of differentially down regulated genes

Gene_id	log2.Fold	pvalue	qvalue	Description
MRA_RS00450	-1.3385	9.59E-18	4.52E-16	hypothetical protein
MRA_RS01285	-1.0123	8.31E-07	1.24E-05	antitoxin
MRA_RS01530	-1.0229	1.81E-05	0.00020064	type VII secretion system protein EsxS
MRA_RS01535	-1.2625	8.99E-09	1.90E-07	ESAT-6-like protein EsxH
MRA_RS01810	-1.161	2.29E-44	5.29E-42	isoniazid-induced protein IniB
MRA_RS02635	-1.1622	1.59E-06	2.20E-05	AURKAIP1/COX24 domain-containing protein
MRA_RS03485	-1.4804	3.99E-08	7.56E-07	antitoxin MazE

MRA_RS03710	-1.0579	4.57E-20	2.58E-18	50S ribosomal protein L4
MRA_RS06365	-1.0928	8.39E-14	2.81E-12	hypothetical protein
MRA_RS08455	-1.0749	5.90E-27	6.52E-25	L-aspartate oxidase
MRA_RS08700	-1.1131	5.29E-06	6.63E-05	50S ribosomal protein L35
MRA_RS09195	-1.1607	1.38E-121	1.17E-118	hypothetical protein
MRA_RS09625	-1.0082	1.02E-16	4.26E-15	hypothetical protein
MRA_RS09970	-1.2471	4.10E-33	7.45E-3	1resuscitation-promoting factor RpfC
MRA_RS10755	-1.0733	3.68E-55	1.04E-52	alpha-crystallin
MRA_RS11920	-1.273	2.64E-32	3.94E-30	beta-ketoacyl-[acyl-carrier-protein] synthase II
MRA_RS15865	-1.0461	2.66E-26	2.81E-24	DNA-binding protein HU
MRA_RS18365	-1.0785	3.22E-08	6.30E-07	30S ribosomal protein S11
MRA_RS18375	-1.1538	6.77E-05	0.00066156	50S ribosomal protein L36
MRA_RS20885	-1.0101	0	0	RNase P RNA component class A
Novel00025	-1.3647	1.29E-06	1.82E-05	
sRNA00004	-1.3697	8.28E-25	7.51E-23	

pvalue is the statistical difference significance test index, q value is corrected pvalue.
